# Quality of life and work productivity and activity impairment among online survey respondents with migraine across a range of headache frequency

**DOI:** 10.3389/fneur.2024.1440733

**Published:** 2024-07-09

**Authors:** Ryotaro Ishii, Fumihiko Sakai, Hiromi Sano, Masami Nakai, Nobuyuki Koga, Miyuki Matsukawa

**Affiliations:** ^1^Kyoto Prefectural University of Medicine, Kyoto, Japan; ^2^Saitama International Headache Center, Saitama Neuropsychiatric Institute, Saitama, Japan; ^3^Medical Affairs, Otsuka Pharmaceutical Co., Ltd., Osaka, Japan; ^4^Medical Affairs, Otsuka Pharmaceutical Co., Ltd., Tokushima, Japan

**Keywords:** migraine burden, Migraine-Specific Quality of Life, WPAI, monthly headache days, claims data, survey

## Abstract

**Objective:**

This study aimed to describe the migraine burden and healthcare utilization in the context of headache frequency using nationwide claims data linked to online survey data previously collected in Japan.

**Background:**

It has been shown that increase in headache frequency can impose greater impact on individuals’ daily and social functioning, but migraine burden in those with low-frequency headaches remains largely unknown in Japan.

**Methods:**

This *post-hoc*, observational study reported on 674 respondents who were working individuals and their family members aged 19–74 years, responded to an online questionnaire (response rate: 14.1% [21,704 responded/153,545 kencom^Ⓡ^ registrants]), and were previously classified as having migraine. Disease burden in terms of Migraine-Specific Quality of Life (MSQ) and Work Productivity and Activity Impairment (WPAI) was compared across 0–3, 4–7, 8–14, and ≥ 15 monthly headache days (MHD).

**Results:**

Among 674 respondents, 419 (62.2%), 148 (22.0%), 61 (9.1%), and 46 (6.8%) had 0–3, 4–7, 8–14, and ≥ 15 MHD, respectively. Of those, 55 (13.1%), 31 (20.9%), 19 (31.1%), and 20 (43.5%) respondents consulted physicians for headaches. Moderate-to-severe impairments in daily activities were reported by 298 (71.1%), 110 (74.3%), 46 (75.4%), and 38 (82.6%) respondents. The proportion of the respondents with WPAI >0% generally increased with increasing headache frequency (presenteeism: 41.7 and 67.5% in respondents with 0–3 and ≥ 15 MHD, respectively; overall work impairment: 44.8 and 72.5%, respectively; and activity impairment: 44.9 and 73.9%, respectively), except for absenteeism (12.4 and 22.5%, respectively). The mean MSQ score declined with increasing MHD (Role function-restrictive: 75.1 and 59.5 in those with 0–3 and ≥ 15 MHD, respectively; Role function-preventive: 85.8 and 75.0, respectively; and Emotional function: 81.9 and 63.6, respectively).

**Conclusion:**

Based on the Japanese nationwide claims data, quality of life and work productivity decreased with increasing numbers of headache days. Substantial disease burden paired with low levels of healthcare utilization highlights the need for medical or non-medical intervention.

## 1 Introduction

Migraine is a primary headache, with global prevalence of 10–15% (1) and some regional variabilities [approximately 15% in the US (2) and Europe (3); 6.0–8.4% in Japan (4, 5)]. This neurological disorder largely affects a wide range of age groups, mainly young and middle-aged individuals (1) in prime working years. Migraine shortens healthy life expectancy; it has been reported that this primary headache is the second leading cause of disability-adjusted life-years among 15 neurological disorders including stroke and dementias (6).

It has been shown that increase in headache frequency can impose greater impact on individuals’ daily and social functioning. Blumenfeld et al. (7), based on a web-based observational study in nine countries including European countries and Taiwan, have compared the impact of migraine on individuals with <15 and ≥ 15 monthly headache days (MHD). The study found that the latter group was associated with significant headache-related disability (measured by the Migraine Disability Assessment [MIDAS]) and impact on health-related quality of life (measured by the Migraine-Specific Quality of Life [MSQ]) compared to those with <15 MHD (7). Additionally, frequency of primary care and specialist visits were significantly higher in those with ≥15 MHD. Furthermore, Doane et al. (8) found, based on the 2017 National Health and Wellness Survey (NHWS) of five European countries, that humanistic and economic burden in terms of including health-related quality of life (measured by the 12-Item Short-Form Health Survey Instrument [SF-12v2]) and Work Productivity and Activity Impairment (WPAI) in ≥4 MHD were greater than those in 1–3 MHD.

In Japan, Kikui et al. (9), based on the 2017 NHWS data, reported that health-related quality of life (SF-12v2) and WPAI, among others, were significantly lower and greater, respectively in respondents with ≥4 MHD than those without migraine. In individuals with low-frequency headaches, however, the migraine burden remains largely unknown. The only data for ≤3 MHD in Japan have been provided by Matsumori et al. (10), in which, based on a nationwide online survey, the authors described burden of migraine (MSQ, migraine interictal burden scale, and WPAI) in people with ≤3 MHD as well as those with ≥4 MHD. In general, the impact increased with an increase in headache frequency.

Previously, Chalmer et al. (11) demonstrated that patients and their relatives with high frequency episodic migraine (HFEM; ≥8 migraine days/month but <15 MHD) who visited Danish Headache Centers did not significantly differ from those with chronic migraine (CM) defined by the International Classification of Headache Disorders, 3rd edition [ICHD-3; i.e., headache occurring ≥15 days/month for >3 months with ≥8 migraine days/month (12)] in terms of the number of migraine attacks and comorbid disease, perceived triptan efficacy, and various sickness benefits. The authors concluded that patients with HFEM were as disabled as those with CM and proposed widening the CM criteria. Comparable results were also reported by Buse et al. (13). Furthermore, a similar line of evidence was provided by Ishii et al. (14), who suggested using headache days instead of migraine days to define CM. Except for the MIDAS scores, individuals with 8–14 MHD had comparable socioeconomic status and experienced a similar degree of burden (e.g., WPAI, pain interference on functioning, and quality of life) to those with 15–23 MHD (14). The authors stated that ≥8 MHD threshold for CM better reflects the burden and disability in people with migraine. Such examination is still scarce, especially in Asian populations.

This study aimed to describe the migraine burden in terms of the MSQ and WPAI, and healthcare utilization in the context of various headache frequencies, including low-frequency headaches (i.e., 0–3, 4–7, 8–14, and ≥ 15 MHD). Additionally, we explored whether the disease burden significantly differed among the MHD categories. This study used a portion of claims data linked to online survey data collected from working individuals across Japan (15). Preceding studies have reported up-to-date epidemiological data, treatment status, and impact on respondents with headache (15, 16).

## 2 Materials and methods

### 2.1 Data source and study population

This *post-hoc*, observational study used medical claims data linked to online questionnaire data collected in an observational, nationwide study on insured health insurance association members aged 19–74 years (15). Several papers have been published on the original data (15–17). The specifics of the original study and its linked data have been provided previously, but briefly, the study used the data linked and anonymized by DeSC Healthcare, Inc. DeSC used the following methods regarding consent to the secondary use of the medical (opt-out opportunities from the DeSC website) and survey data (604,102 people). For the survey data, in the preface of the questionnaire on the kencom^Ⓡ^ app, an explanation was provided to its registrants regarding the secondary use of survey responses. Proceeding with the questionnaire indicates consent for the secondary use. The study participants were therefore those who registered in the health promotion support service application kencom^Ⓡ^ (153,545 people) and responded to an online health-related questionnaire survey administered by DeSC Healthcare, Inc. between 1 and 30 November 2020 (21,704 people). We then excluded individuals whose age and sex data in the questionnaire responses differed from those found in the claims data to ensure the reliability of the response data (resulting in 21,480 people with and without headaches). Among these, 7,311 individuals self-reported “having experienced headaches in the past 3 months,” and 691 were classified as having migraine based on the questionnaire responses in accordance with ICHD-3 (15, 16). Finally, this study focuses on 674 respondents with migraine whose medical data were available at least 1 year prior to the month of the survey (i.e., data available from December 2019 or earlier).

### 2.2 Ethics statement

This study used secondary anonymized data, and no additional individual-level consent was obtained for data usage. This study was approved by the independent ethics committee of Otsuka Pharmaceutical Co., Ltd. (approval No.: RI221012) and was registered (UMIN000050351). The study protocol of the original observational study that used medical claims data linked to online questionnaire data (15) was approved by the ethics committee of the Research Institute of Healthcare Data Science (approval No.: RI2021005). The survey was conducted in accordance with the Ethical Guidelines for Medical and Biological Research Involving Human Subjects in Japan and the Declaration of Helsinki (revised in October 2013). The authors have full access to the study data.

### 2.3 Background characteristics

The extracted sociodemographic and clinical characteristics of the respondents included sex, age, employment status, annual household income, years lived with headache, comorbidities potentially affecting headache (identified from 1 December 2019 to 30 November 2020), Charlson Comorbidity Index (CCI) (18, 19) (identified in the same time period as for comorbidities), and triptan prescriptions (identified from 1 June to 30 November 2020). The examined comorbidities were epilepsy, hypertension, cardiovascular disorders, cerebrovascular disorders, schizophrenia, anxiety disorders, somatoform disorders, depression, sleep disorders, asthma, gastrointestinal disorders, allergies, and autoimmune disorders. The disease codes for these comorbidities in the International Classification of Diseases 10th revision are provided in Table 1 and Supplementary Table 1.

**Table 1:** Sociodemographic and clinical characteristics of survey respondents.

Characteristics	Total		Monthly headache days							
			0–3		4–7		8–14		≥15	
	(*N* = 674)		(*n* = 419)		(*n* = 148)		(*n* = 61)		(*n* = 46)	
	*n*	(%)	*n*	(%)	*n*	(%)	*n*	(%)	*n*	(%)
Sex*										
Male	265	(39.3)	154	(36.8)	71	(48.0)	25	(41.0)	15	(32.6)
Female	409	(60.7)	265	(63.2)	77	(52.0)	36	(59.0)	31	(67.4)
Age*										
Mean (SD)	43.2	(8.8)	43.5	(8.6)	43.0	(9.1)	42.7	(9.0)	42.2	(8.4)
Median (min, max)	44.0 (24, 65)		44.0 (24, 65)		44.0 (24, 61)		42.0 (26, 63)		41.5 (26, 60)	
19–29	50	(7.4)	29	(6.9)	15	(10.1)	5	(8.2)	1	(2.2)
30–39	179	(26.6)	110	(26.3)	33	(22.3)	19	(31.1)	17	(37.0)
40–49	260	(38.6)	165	(39.4)	59	(39.9)	21	(34.4)	15	(32.6)
50–59	175	(26.0)	110	(26.3)	38	(25.7)	15	(24.6)	12	(26.1)
60–74	10	(1.5)	5	(1.2)	3	(2.0)	1	(1.6)	1	(2.2)
Employment status										
Employed	584	(86.6)	362	(86.4)	129	(87.2)	54	(88.5)	39	(84.8)
Unemployed	90	(13.4)	57	(13.6)	19	(12.8)	7	(11.5)	7	(15.2)
Annual household income										
<¥1,000,000	10	(1.5)	7	(1.7)	1	(0.7)	0	(0.0)	2	(4.3)
¥1,000,000 to <¥5,000,000	128	(19.0)	80	(19.1)	27	(18.2)	13	(21.3)	8	(17.4)
¥5,000,000 to <¥10,000,000	356	(52.8)	218	(52.0)	78	(52.7)	32	(52.5)	28	(60.9)
≥¥10,000,000	118	(17.5)	71	(16.9)	32	(21.6)	11	(18.0)	4	(8.7)
Do not know	51	(7.6)	36	(8.6)	8	(5.4)	3	(4.9)	4	(8.7)
No answer	11	(1.6)	7	(1.7)	2	(1.4)	2	(3.3)	0	(0.0)
Years lived with headache										
<11	104	(15.4)	60	(14.3)	28	(18.9)	4	(6.6)	12	(26.1)
11–20	106	(15.7)	56	(13.4)	24	(16.2)	16	(26.2)	10	(21.7)
≥21	141	(20.9)	70	(16.7)	37	(25.0)	21	(34.4)	13	(28.3)
Do not remember or no answer	323	(47.9)	233	(55.6)	59	(39.9)	20	(32.8)	11	(23.9)
Comorbidities*										
0**	380	(56.4)	243	(58.0)	85	(57.4)	33	(54.1)	19	(41.3)
1**	126	(18.7)	78	(18.6)	32	(21.6)	9	(14.8)	7	(15.2)
≥2**	168	(24.9)	98	(23.4)	31	(20.9)	19	(31.1)	20	(43.5)
Depression	43	(6.4)	21	(5.0)	8	(5.4)	4	(6.6)	10	(21.7)
Anxiety disorder	28	(4.2)	12	(2.9)	8	(5.4)	6	(9.8)	2	(4.3)
Sleep disorder	55	(8.2)	24	(5.7)	8	(5.4)	10	(16.4)	13	(28.3)
CCI*										
Mean (SD)	0.3	(0.8)	0.3	(0.7)	0.2	(0.4)	0.5	(1.1)	0.7	(1.5)
Median (min, max)	0 (0, 9)		0 (0, 5)		0 (0, 2)		0 (0, 7)		0 (0, 9)	
Prophylaxis*										
No	620	(92.0)	398	(95.0)	134	(90.5)	53	(86.9)	35	(76.1)
Yes	54	(8.0)	21	(5.0)	14	(9.5)	8	(13.1)	11	(23.9)
Types of prophylaxis*										
1	48	(7.1)	20	(4.8)	14	(9.5)	6	(9.8)	8	(17.4)
> = 2	6	(0.9)	1	(0.2)	0	(0.0)	2	(3.3)	3	(6.5)
Triptan prescribed per month*, tablet										
*n*	40	(5.9)	8	(1.9)	17	(11.5)	8	(13.1)	7	(15.2)
Mean (SD)	12.5	(7.2)	9.8	(5.7)	11.0	(4.2)	15.7	(7.2)	15.6	(12.2)
Median (min, max)	10.0 (2, 36)		10.0 (3, 20)		10.0 (5, 20)		11.8 (10, 26.7)		10.0 (2, 36)	
Triptan prescribed ≥10 tablets per month*										
Yes	27	(67.5)	5	(62.5)	10	(58.8)	8	(100.0)	4	(57.1)

*Data were extracted from medical claims data. Comorbidities and CCI were identified from 1 December 2019 to 30 November 2020, and triptan and prophylactic prescriptions were identified from 1 June to 30 November 2020. Unless otherwise stated, other information was obtained at the time when a questionnaire was answered.

**The examined comorbidities that affect headache were epilepsy (International Classification of Diseases 10th revision codes: G40 and G41), hypertension (I10–I15), cardiovascular disorders (I20–I28, I30–I52), cerebrovascular disorders (I60–I69), schizophrenia (F20), anxiety disorders (F40 and F41), somatoform disorders (F45), depression (F32 and F33), sleep disorders (G47), asthma (J45 and J46), gastrointestinal disorders (K20–K31, K35–K38, K40–K46, K50–K52, K55–K67, K70–K77, K80–K87, and K90–K93), allergies (J30, L23, J450, K522, L500, and T784), and autoimmune disorders (codes listed in Supplementary Table 1).

SD, standard deviation; min, minimum; max, maximum; CCI, Charlson Comorbidity Index.

Regarding healthcare utilization, data on physician consultation for headaches or migraine in the past 6 months and prophylactic use of antidepressants, anti-epileptics, calcium channel blockers, angiotensin-receptor blockers/angiotensin converting enzyme inhibitors, beta-blockers, and others from 1 June 2020 to 30 November 2020 were extracted from the questionnaire responses and claims data, respectively.

### 2.4 Outcome measures

The MSQ Questionnaire version 2.1 (20) was used to compare the quality of life among the respondents. The MSQ is a 14-item patient-reported outcome instrument that measures the impact of migraine across three aspects of quality of life (Role function-restrictive [RR], Role function-preventive [RP], and Emotional function [EF]) over the past 4 weeks (21, 22). Higher scores indicate a better quality of life.

The WPAI Questionnaire-General Health was used to compare general health and symptom severity in terms of work productivity and regular activities among the respondents. The WPAI is expressed as the percentage of work time missed due to headache (absenteeism), degree of impairment while working due to headache (presenteeism), degree of overall work impairment due to headache (overall work impairment), and degree of activity impairment due to headache (activity impairment) during the past 7 days (23). Higher scores indicate greater work and activity impairment.

### 2.5 Statistical analysis

The background characteristics of the survey respondents and outcome measures were descriptively summarized for the total population (*N* = 674) and each MHD category of 0–3, 4–7, 8–14, ≥15 MHD, with mean (standard deviation [SD]), median (minimum, maximum), and frequency and percentage. The categories were selected to understand different degrees of migraine burden in low to high MHD, and the same categories were also used in previous studies based on recent convention (10, 13). The MHD category for each respondent was determined based on the questionnaire responses regarding headache frequency in the past month (0–30 MHD).

The relationship between the MHD and MSQ scores was examined using a scatter plot, and the Pearson correlation coefficient was estimated. To compare MSQ scores across the MHD categories, we used analysis of covariance, with the covariates of sex, age, comorbidities (depression, anxiety disorder, and sleep disorder (24)), CCI, and triptan prescription of ≥10 tablets/month, according to previous studies (7, 8, 11). After examining the distribution of values in the histogram, it was concluded that assuming normal distribution posed no issue. In the WAPI analysis across the MHD categories, the WPAI score was converted into a dichotomous variable (0% or > 0%) because of the large proportion of respondents with a WPAI score of zero. We used multiple logistic regression models with the same covariates as used in the analysis for MSQ. To explore where the score differences exist among the categories of headache days (0–3, 4–7, 8–14, ≥15 MHD), we conducted contrast test [(−3, 1, 1, 1), (−1, −1, 1, 1), (−1, −1, −1, 3)] (i.e., 0–3 vs. ≥4 MHD, 0–7 vs. ≥8 MHD, and 0–14 vs. ≥15 MHD) based on the above analysis of covariance and logistic regression models. We did not separate the last group into subcategories (i.e., 0–23 vs. ≥24 MHD), because an analysis using the finer subcategories on our data is statistically vulnerable and we considered it compromise the reliability of the results. As the database population of the present study was closer to the general population in Japan, there were few participants with 15 or more MHD compared with the population of Ishii et al. (14). The mean MSQ score differences or odds ratios (95% lower and upper confidence limits) between the two MHD categories were also estimated.

All available data were used in this study, and no imputation was performed for missing data. No statistical power calculation was conducted prior to the study. The sample size was selected according to available data. The statistical tests were conducted at a significance level *p* < 0.05 (two-sided), and confidence intervals were estimated at a 95% confidence level. Data were analyzed using SAS Release 9.4 (SAS Institute, Inc., NC, USA).

## 3 Results

### 3.1 Sociodemographic and clinical characteristics

Of the 674 respondents with migraine, up to two-thirds reported 0–3 MHD (62.2%, *n* = 419), and 148 (22.0%), 61 (9.1%), and 46 (6.8%) respondents reported 4–7, 8–14, and ≥ 15 MHD, respectively (Table 1). The distribution of sex and age was relatively comparable across the MHD categories. The majority of the respondents across the MHD categories were employed (86.4% [362/419], 87.2% [129/148], 88.5% [54/61], and 84.8% [39/46] in 0–3, 4–7, 8–14, and ≥ 15 MHD, respectively). The proportions of the respondents living with headache for ≥11 years were 30.1% (126/419), 41.2% (61/148), 60.7% (37/61), and 50.0% (23/46) in 0–3, 4–7, 8–14, and ≥ 15 MHD, respectively. Although the CCI scores (calculated based on the fixed comorbidities contributing to mortality) were relatively similar across the MHD categories, the proportion of respondents with ≥2 selected comorbidities (potentially affecting headache; Table 1 and Supplementary Table 1) increased with increasing headache frequency: 23.4% (98/419) with 0–3 MHD and 43.5% (20/46) with ≥15 MHD.

**Figure 1:**
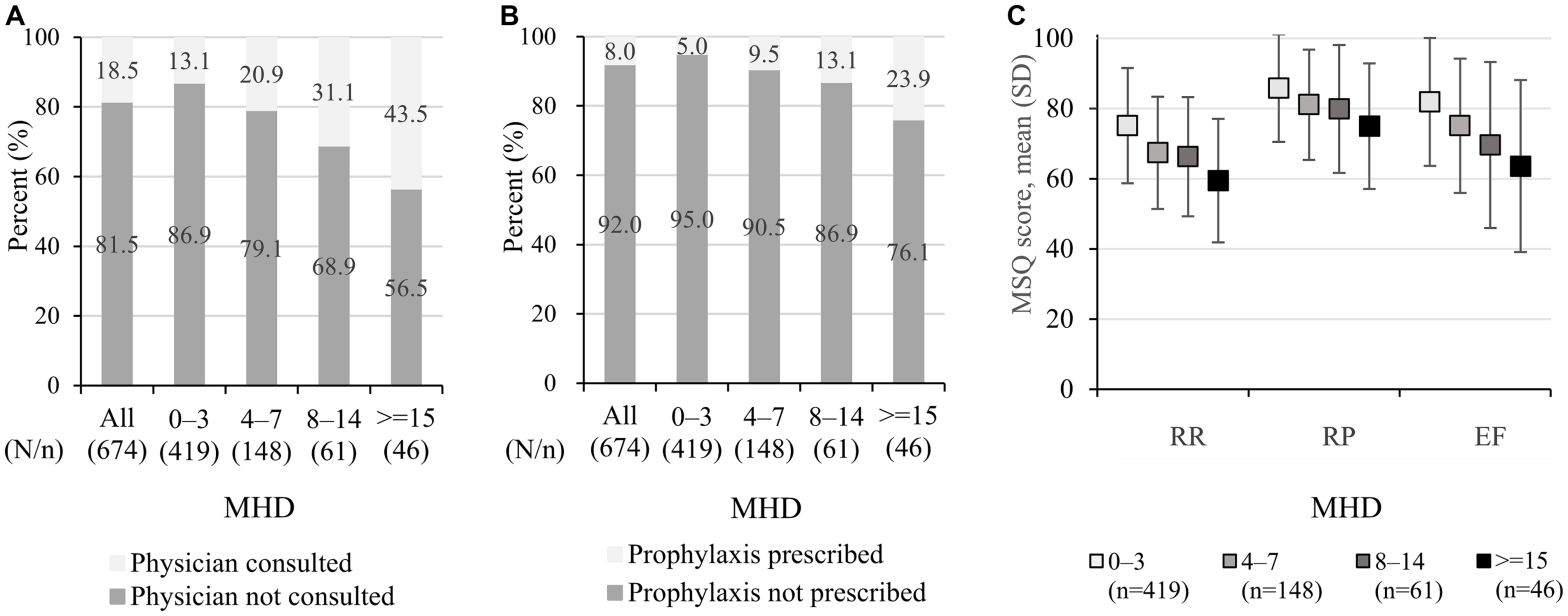
Healthcare utilization and MSQ: **(A)** physician consultation, **(B)** prophylaxis use, and **(C)** MSQ score. **(A)** The data show physician consultation for headaches or migraine in the past 6 months before taking part in the questionnaire and were extracted from the questionnaire responses. **(B)** The data show prescribed prophylactics (antidepressants, anti-epileptics, calcium channel blockers, angiotensin-receptor blockers/angiotensin converting enzyme inhibitors, beta-blockers, and others) and were extracted from medical claims data, and the identification period was from 1 June 2020 to 30 November 2020. **(C)** The data shown represent mean +/− standard deviation. MSQ, migraine-specific quality of life; MHD, monthly headache days; RR, role function-restrictive; RP, role function-preventive; EF, emotional function.

The proportion of the respondents who consulted physicians in the past 6 months were low overall (18.5% [125/674]) but increased with increasing MHD (13.1% [55/419] for 0–3 and 43.5% [20/46] for ≥15 MHD, respectively; Figure 1A). Likewise, prophylaxis use in the past 6 months was low in general (8.0% [54/674]), with slight increase with increasing MHD (5.0% [21/419] for 0–3 and 23.9% [11/46] for ≥15 MHD, respectively; Figure 1B).

### 3.2 MSQ and WPAI

Among 674 respondents, the MSQ scores for all three aspects were relatively high in the 0–3 MHD category, and the scores declined with increasing MHD. The mean (SD) RR scores were 75.1 (16.4), 67.4 (16.0), 66.3 (17.0), and 59.5 (17.6) in 0–3, 4–7, 8–14, and ≥ 15 MHD (Figure 1C). Similarly, the mean scores of RP were 85.8 (15.3), 81.1 (15.7), 79.9 (18.2), and 75.0 (17.9) for 0–3, 4–7, 8–14, and ≥ 15 MHD, respectively, and those of EF were 81.9 (18.2), 75.1 (19.1), 69.6 (23.6), and 63.6 (24.5), respectively. A scatter plot of the MHD and MSQ scores is provided in Supplementary Figure 1.

**Figure 2:**
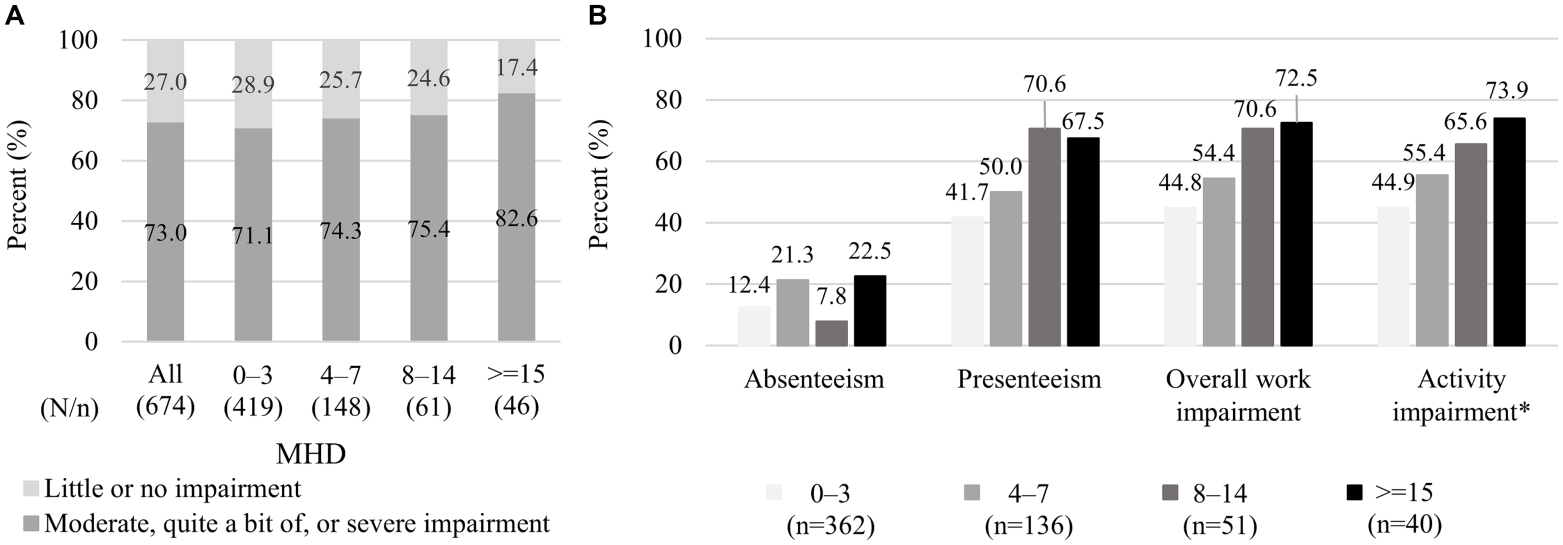
Impairment in daily activities and work: **(A)** level of impairment in daily activities and **(B)** the proportion of respondents with WPAI > 0% in each WPAI aspect. **(A)** The data shown represent the degree of impairment when not taking medicines. The data were extracted from questionnaire responses to the question “How much does a single headache impact your daily life?,” and each respondent selected an answer from five answers of no, little, moderate, quite a bit, or severe impairment. **(B)** The data shown exclude respondents with a WPAI score zero and the corresponding number of respondents is shown in brackets. * For absenteeism, presenteeism, and overall work impairment, data were unavailable from individuals who responded “no” to the question “Are you currently working (in the position that involves wages)?,” whereas responses for activity impairment were obtained from all individuals (*n* = 419, 148, 61, and 46 in 0–3, 4–7, 8–14, and ≥ 15 MHD categories, respectively). WPAI, Work Productivity Activity Impairment; MHD, monthly headache days.

Among 674 respondents, moderate, quite a bit of, or severe impairments in daily activities were reported by 73.0% (492/674) of the overall respondents, and the percentage was high even in the 0–3 MHD category (71.1% [298/419]) and slightly increased with increasing MHD (82.6% [38/46] in ≥15 MHD; Figure 2A). As for WPAI, all data from 674 respondents were available for activity impairment, whereas the data for absenteeism, presenteeism, and overall work impairment were unavailable from non-working respondents but were available from 589 working individuals. The WPAI absenteeism, the percentages of the respondents with WPAI >0% (i.e., >0% of work time missed due to headache) were 12.4% (45/362), 21.3% (29/136), 7.8% (4/51), and 22.5% (9/40) in 0–3, 4–7, 8–14, and ≥ 15 MHD, respectively (Figure 2B). For presenteeism, overall work impairment, and activity impairment, the percentages of the respondents with WPAI >0% (i.e., some impairment while working or engaging in daily activities due to headache) for each MHD category were as follows: 41.7% (151/362), 50.0% (68/136), 70.6% (36/51), and 67.5% (27/40); 44.8% (162/362), 54.4% (74/136), 70.6% (36/51), and 72.5% (29/40); and 44.9% (188/419), 55.4% (82/148), 65.6% (40/61), and 73.9% (34/46), respectively. The proportions of WPAI >0% were similarly high for 8–14 and ≥ 15 MHD in all three WPAI subitems.

### 3.3 MSQ and WPAI differences among MHD categories

**Figure 3:**
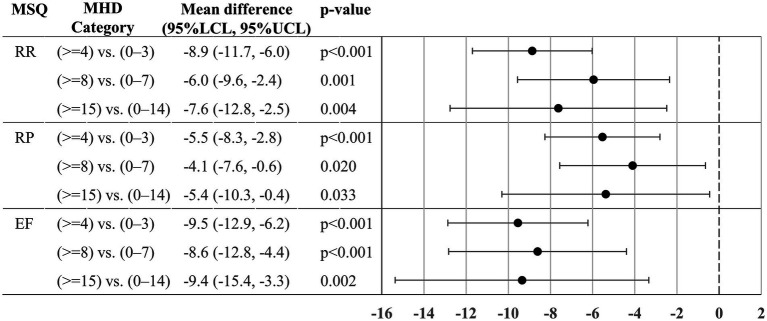
Mean MSQ score difference between MHD categories. The MSQ score differences (95% confidence intervals) for the two MHD categories were estimated by the analysis of covariance. The model (*N* = 674) included sex, age, comorbidities (depression, anxiety disorder, and sleep disorder), Charlson Comorbidity Index, and triptan prescription of ≥10 tablets/month as covariates. For each comparison, we treated a lower MHD category as a reference in the analysis (e.g., 0–3 MHD, 0–7 MHD, 0–14 MHD). MSQ, migraine-specific quality of life; MHD, monthly headache days; LCL, lower confidence limit; UCL, upper confidence limit; RR, Role function – restrictive; RP, Role function – preventive; EF, Emotional function.

All contrast tests showed that the adjusted MSQ scores were significantly lower in the higher MHD categories (more severe) than in the lower categories (Figure 3). The mean (95% lower confidence limit, 95% upper confidence limit) RR score difference between 0 and 3 vs. ≥4 MHD was −8.9 (−11.7, −6.0), and the difference between 0 and 7 vs. ≥8 MHD and 0–14 vs. ≥15 MHD was −6.0 (−9.6, −2.4) and −7.6 (−12.8, −2.5), respectively. Comparable results were obtained for the other two MSQ aspects.

**Figure 4:**
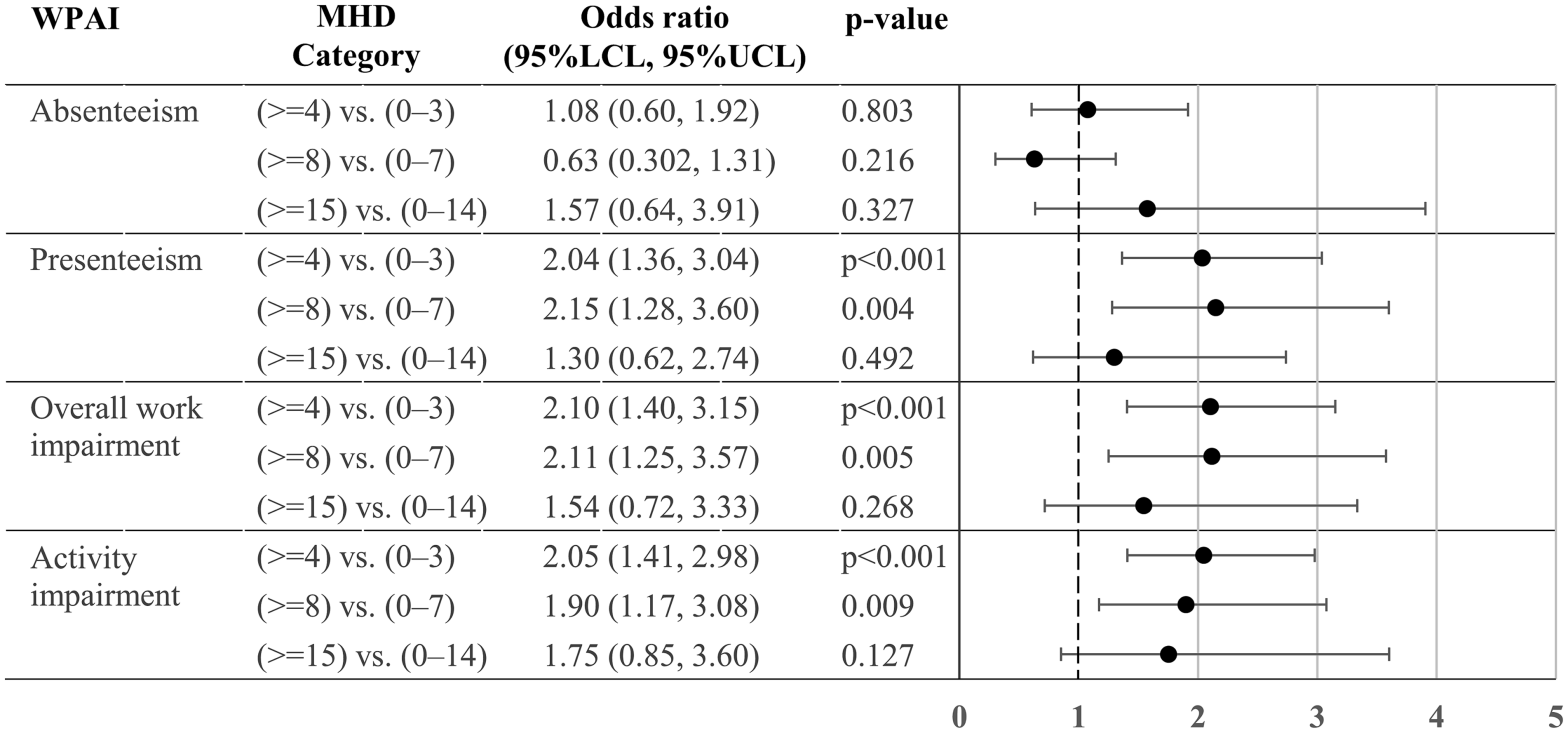
Odds ratio for the WPAI > 0% between MHD categories. The odds ratios (95% confidence intervals) of WPAI >0% for the two MHD categories were estimated by a logistic regression model. The model (*n* = 589 for absenteeism, presenteeism and overall work impairment; and *N* = 674 for activity impairment) included sex, age, comorbidities (depression, anxiety disorder, and sleep disorder), Charlson Comorbidity Index, and triptan prescription of ≥10 tablets/month as covariates. For absenteeism, presenteeism, and overall work impairment, data were unavailable from individuals who responded “no” to the question “Are you currently working (in the position that involves wages)?.” For each comparison, we treated a lower MHD category as a reference in the analysis (e.g., 0–3 MHD, 0–7 MHD, 0–14 MHD). WPAI, Work Productivity Activity Impairment; MHD, monthly headache days; LCL, lower confidence limit; UCL, upper confidence limit.

For the analysis for activity impairment, data from 674 respondents were available, whereas that of the other WPAI components was conducted based on data from 589 working individuals. The adjusted odds of presenteeism >0% (i.e., some impairment while working due to headache) were significantly higher in ≥4 MHD (vs. 0–3) and ≥ 8 MHD (vs. 0–7), but no statistical difference was observed between 0 and 14 vs. ≥15 MHD (Figure 4). Similarly, there were no significant differences between 0 and 14 vs. ≥15 MHD in overall work impairment and activity impairment. The odds of absenteeism >0% (i.e., >0% of work time missed due to headache) were similar between 0 and 3 vs. ≥4 MHD, 0–7 vs. ≥8 MHD, and 0–14 vs. ≥15 MHD.

## 4 Discussion

This study described the range of migraine burden across four headache frequency subgroups in 674 respondents who were classified as having migraine in a previous nationwide observational study on the members of health insurance associations in Japan (15) (of which, approximately 90% were workers). In this study, we found that as MHD increased, the disease burden in terms of MSQ and WPAI increased, which is generally in line with previous reports (10, 14, 25). Additionally, MSQ scores were significantly different for all contrast tests of the two MHD categories. The odds of WPAI >0% were significantly higher in participants with at least 4 MHD (vs. 0–3) and 8 MHD (vs. 0–7) categories in all WPAI aspects except for absenteeism, whereas those with at least 15 MHD were similar to those with 0–14 MHD. Regardless of the statistical significance, in general, the incremental increase in both MSQ and WPAI may imply that all frequency categories sufficiently capture different degrees of migraine burden.

Our data showed that the burden of migraine was present even among respondents with low-frequency headaches. Of those with 0–3 MHD, approximately 71% when not taking medicines reported moderate to severe impairment in daily activities and two-thirds (excluding those who responded “Do not remember” or no answer) reported headaches lasting 11 years or longer. Furthermore, around 42% of the respondents reported work productivity impairment (presenteeism) to some extent, and 45% reported total work impairment (overall work impairment). Similarly, 45% of the respondents reported that leisure time was negatively impacted as well as work time. Yet, only around 13% of the 0–3 MHD subgroup sought medical attention and 5% used prophylaxis. Individuals with this low-frequency headache comprised around 62% of our study population, and a similarly large proportion was found in a previous population-based web survey in Japan (approximately 67% of the migraine population) (10). Despite the large number of migraine patients with 0–3 MHD and the high disease burden, these results suggest that a large number of people are potentially left unattended. Additionally, these patients have not been well studied (9, 26). Further studies are required to identify interventions that could help improve the health conditions of those affected.

Regardless of the headache frequency, the overall consultation rate was low (approximately 19%). These results were in line with a population survey of IT employees by Shimizu et al. (27) in Japan. Similarly, little use of prophylaxis, as observed in our study in 8% of the respondents, was also reported in previous studies. A study using an employment-based Japanese claims database reported that preventive treatment for migraine was rarely used, even among migraine patients treated at medical institutions (28). Matsumori et al. (10) also found comparable results for preventive treatments (72.5% were employed), although the consultation rates in the previous year were much higher (39.7%) than those found in our study. Lipton et al. (25) found that 40.4% of their study respondents with migraine were determined to be eligible for preventive medications based on headache frequency and MIDAS grade. Furthermore, over 81.5% of the respondents with ≥4 MHD were eligible for preventive medications (25). Altogether, these results suggest that treatment access, especially in individuals with moderate to high headache frequency (e.g., ≥4 MHD), requires further improvement. It has been suggested that understanding factors influencing the migraine stigma (e.g., personal and workplace stigma) could help improve healthcare access (29). Raising awareness through disease education for both workers with and without headaches may help improve access to medical care and suitable treatments.

We found significant MSQ differences between the two categories in all contrast tests, whereas for work and activity impairment, we found a significant difference in the proportion of WPAI 0 and > 0% (some degree of impairment) between 0 and 7 and ≥ 8 MHD (except for absenteeism) but did not find a difference between 0 and 14 and ≥ 15 MHD (Figure 4). Previously, Ishii et al. (14) demonstrated, using the American Registry for Migraine Research, that functional disability in terms of, among others, the WPAI were not substantially different between 8 and 14 and 15–23 MHD. The authors proposed ≥8 MHD threshold to distinguish CM from episodic migraine instead of ≥15 MHD. Based on our WPAI results, we also did not find evidence supporting the ≥15 MHD threshold to classify CM.

To underscore the similarity of the results derived from the two studies, we note here some differences between them. First, Ishii et al. (14) compared the burden among four MHD categories (i.e., 0–7, 8–14, 15–23, and ≥ 24 MHD), whereas we did so more broadly between the two groups (i.e., 0–3 vs. ≥4, 0–7 vs. ≥8, or 0–14 vs. ≥15 MHD). Thus, individuals with a very high headache frequency (e.g., ≥24 MHD) were not examined separately from those with ≥15 frequency. Additionally, we used binary WPAI (0% or > 0%). Second, the ARMR study recruited participants at specialty headache clinics, and therefore a larger proportion of the study population had higher headache frequency (44.3% categorized in ≥15 MHD, as opposed to 6.8% in our study). Furthermore, 58.1% of the participants in the former study were employed, as opposed to 86.6% employed in our study. Other differences such as sex ratio and race were also observed. As migraine has both common [e.g., age range (4), familial aggregation (30), and trigger or triggered by other disorders (31–33)] and different features [e.g., prevalence (1–5)] presumably due to genetic predispositions and cultural (34) or socioeconomic backgrounds, it is notable that a similar tendency was observed in our study using nationwide survey data in Japan. Further studies on cut-offs that better reflect the burden of CM, other outcome measures, and other geographic regions are indeed of clinical value.

### 4.1 Limitations

As previously described in Sakai et al. (15), our results may not be generalizable to the entire Japanese population with migraine, because the study participants were employees and their family members of employment-based health insurance associations (86.6% were employed). This population possibly has relatively high socioeconomic status compared to the general population. Additionally, the study participants were recruited from the registrants of kencom^Ⓡ^ app. The registrants generally have a slightly higher male representation and a higher level of health consciousness than non-kencom^Ⓡ^ registrants, and they also have access to the internet. As aforementioned, severe migraine cases may have been less represented in our study. Additionally, it is unknown whether cases of medication overuse headache are included in our study population. As some medicines such as non-steroidal anti-inflammatory drugs can be obtained through both prescription and over-the-counter in Japan, complete information on medications taken for acute and/or symptomatic treatment of headache is not recorded in the database. Nonetheless, the proportion of individuals with 0–3 MHD was comparable to that reported in other population-based studies (10, 13). Some questionnaire responses, including the MSQ (past 4 weeks) and WPAI (past 7 days), were self-reported and were subject to self-report bias and recall bias, whereas such bias was not present in the claims data, which were subject to classification and entry errors. To statistically test WPAI among MHD categories, WPAI data were transformed into a binary variable (0% or > 0%) owing to excessive zeros. Additionally, we used adjusted statistical models to compare MSQ and WPAI among the MHD categories; however, not all confounding variables could be incorporated into the models, and it is possible that our results were potentially confounded. Therefore, the results should be interpreted with caution. Of note, our assessments regarding prophylaxis use did not include monoclonal antibodies, as they were launched recently in Japan after the questionnaire administration. Lastly, our study population with migraine was classified based on self-reported questionnaire responses, and it is unknown whether these were true migraine cases. Mindful of the limitations, the data represent findings from a relatively large number of survey respondents across Japan.

## 5 Conclusion

Using nationwide healthcare claims data linked to online survey data, this study found a frequency-dependent increase in the migraine burden. Additionally, the majority of the respondents, even those with low-frequency headache, reported moderate to severe impairment in daily activities, and except for absenteeism some degree of work and activity impairment was observed in 41–74% of the respondents. Despite the substantial disease burden, physician consultation rates and prophylactic use were low. Our study highlights the need for intervention, either medical or non-medical, in patients with higher headache frequency and in those with lower frequency, who have so far received relatively little attention.

## Data availability statement

The data analyzed in this study is subject to the following licenses/restrictions: the data that support the findings of this study are available from DeSC Healthcare, Inc. (Tokyo, Japan) but restrictions apply to the availability of these data, which were used under license for the current study, and so are not publicly available. Data are however available from the authors upon reasonable request and with permission of DeSC Healthcare, Inc. Requests to access these datasets should be directed to MM, Matsukawa.Miyuki@otsuka.jp.

## Ethics statement

The studies involving humans were approved by the Independent Ethics Committee of Otsuka Pharmaceutical Co., Ltd. (Approval number: RI221012). The studies were conducted in accordance with the local legislation and institutional requirements. Written informed consent from the patients/participants or patients/participants’ legal guardian/next of kin was not required to participate in this study in accordance with the national legislation and the institutional requirements.

## Author contributions

RI: Conceptualization, Methodology, Writing – review & editing. FS: Conceptualization, Methodology, Writing – review & editing. HS: Conceptualization, Funding acquisition, Methodology, Project administration, Writing – review & editing. MN: Conceptualization, Methodology, Writing – review & editing. NK: Conceptualization, Methodology, Writing – review & editing. MM: Conceptualization, Formal analysis, Methodology, Writing – review & editing.
