# Supplementary material for: Quality of life and work productivity and activity impairment among online survey respondents with migraine across a range of headache frequency
Source: Front Neurol. 2024 Jul 9;15:1440733. doi: 10.3389/fneur.2024.1440733 (PMC11263292; doi:10.3389/fneur.2024.1440733)
Supplement: Supplementary file 1 [file Data_Sheet_1.PDF]

Supplementary Material

Supplementary Table 1 A list of autoimmune disorders

**Disease**	**International Classification of Diseases 10th revision codes**
Diabetes mellitus, insulin dependent	E10
Autoimmune thyroid disease	E035<br>E039<br>E050<br>E055<br>E059<br>E063<br>E065
Autoimmune polyglandular syndrome	E310
Reactive arthritis (Reiter's syndrome)	M023<br>M028<br>M029
Rheumatoid arthritis	M05<br>M06<br>M080<br>M081<br>M082<br>M083<br>M084
Ankylosing spondylitis	M45
Polyarteritis nodosa and related condition<br>(Incl. Kawasaki, Churg-Strauss syndrome, etc.)	M30
Thrombotic microangiopathy	M311
Granulomatosis with polyangiitis (Wegeners's nulomatosis)	M313
Microscopic polyangiitis	M317
Henoch-Schonlein purpura	D690
Giant cell arteritis/ Polymyalgia rheumatica	M353<br>M315<br>M316
Systemic lupus erythematosus	M32
Polymyositis/dermatomyositis	M330<br>M331<br>M332<br>M339
Systematic sclerosis (scleroderma)	M34
Sjögren's syndrome	M350
Mixed connective tissue disease	M351
Behcet's syndrome	M352
Pemphigus vulgaris	L100

**Disease**	**International Classification of Diseases 10th revision codes**
Bullous pemphigoid	L12
Dermatitis herpetiformis	L130
Psoriasis	L40
Alopecia areata	L64
Vitiligo	L80
Pernicious anemia	D510
Autoimmune hemolytic anemia	D590<br>D591
Idiopathic thrombocytopenic purpura	D693
Acute disseminated encephalomyelitis	G04
Anti-NMDA receptor encephalitis	G131
Multiple sclerosis	G35
Neuromyelitis optica and ADEM	G36
Guillain-Barré syndrome	G610<br>G611<br>G618<br>G61.9
Myasthenia gravis	G700
Primary biliary cirrhosis	K743
Crohn's disease	K50
Ulcerative colitis	K51
Coeliac disease	K900
Acute rheumatic fever and chorea	I00<br>I010<br>I011<br>I012<br>I018<br>I019<br>I020<br>I029
Sarcoidosis	D86
IgA nephropathy	N00<br>N01<br>N03<br>N05

Supplementary Figure 1 A scatter plot between MHD and MSQ scores

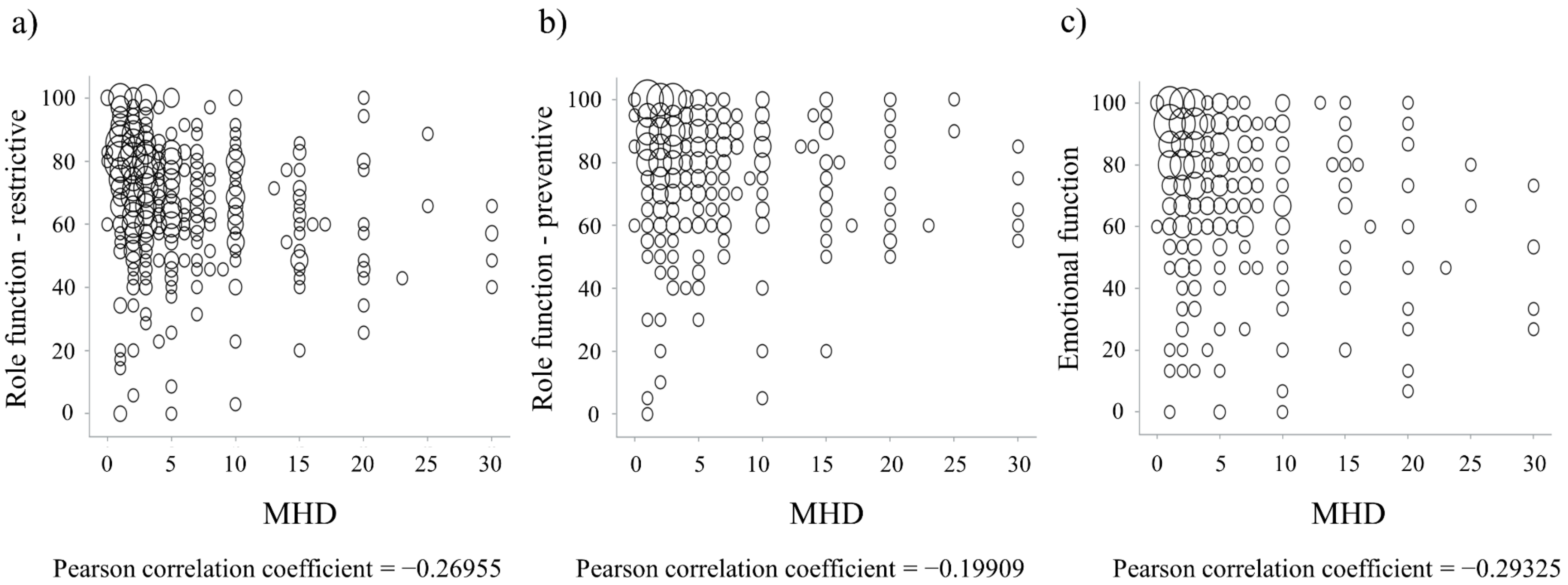

Abbreviations: MHD, monthly headache days; MSQ, migraine-specific quality of life.
